# 980. *Enterobacter*: An Early-Onset Pathogen of Prosthetic Hip and Knee Infections with Poor Prognoses

**DOI:** 10.1093/ofid/ofac492.822

**Published:** 2022-12-15

**Authors:** Isabel P Prado, Billy I Kim, Andrew Schwartz, Colleen M Wixted, Breanna Polascik, Edward Hendershot, William A Jiranek, Jessica Seidelman, Thorsten M Seyler

**Affiliations:** Duke University, Durham, North Carolina; Duke University, Durham, North Carolina; Duke University Medical Center, Durham, North Carolina; Duke University, Durham, North Carolina; Duke University School of Medicine, Durham, North Carolina; Duke University Medical Center, Durham, North Carolina; Duke University, Durham, North Carolina; Duke University School of Medicine, Durham, North Carolina; Duke University, Durham, North Carolina

## Abstract

**Background:**

*Enterobacter sp.* is a gram-negative, often drug-resistant bacillus that is classically identified from mucosal surfaces, such as pulmonary or urinary tracts. Despite its virulence, *Enterobacter* represents a less common pathogen in prosthetic joint infections (PJI). This study aims to resolve the current paucity of described clinical characteristics and treatment success of *Enterobacter* PJI.

**Methods:**

Thirty-three patients with *Enterobacter aerogenes-, cloaecae/asburiae-*, or *hormachei*-positive PJIs were identified at a single institution. Infection clearance was defined as absence of infection or repeat surgery at one year and off all adjunct antibiotic therapy. Patient demographics, PJI characteristics, treatments, and clinical outcomes were compared between patients with and without clearance.

**Results:**

In this cohort, mean age was 66.8 years (SD: 11.6), mean BMI was 31.0 (SD: 7.1), and mean Elixhauser Comorbidity Score was 9.1 (SD: 4.5). 54.5% had a prior PJI in the same joint, and 87.9% had more than one prior surgery in that joint. Median time of PJI diagnosis after surgery was 22 days [IQR: 18-60]. Patients initially treated with debridement antibiotics and implant retention (n=11) had a 45.5% clearance rate, compared to 23.1% for 2-stage exchange (n=13) and 44.4% for those requiring resection arthroplasty or amputation (n=9). With a median follow-up of 2.9 years, final joint outcomes included resection arthroplasty in 9 (27.3%), above-knee amputation in 7 (21.2%), definitive treatment with an articulating spacer in 6 (18.2%), and arthrodesis in 2 (6.1%) patients.

**Conclusion:**

*Enterobacter* PJI often presents acutely and is a difficult pathogen to treat. The outcomes of our cohort reflect the virulence of *Enterobacter*, with high rates of failure and ultimate joint- or limb-sacrificing procedure. Despite being a less common cause of PJI, prognosis is exceptionally poor with traditional treatment algorithms when compared to historical cohorts of more common pathogens.

**Disclosures:**

**William A. Jiranek, MD**, Biomech Holdings LLC: Stocks/Bonds|DePuy, A Johnson & Johnson Company: IP royalties **Thorsten M. Seyler, MD, PhD**, Heraeus: Paid consultant|Pattern Health: IP royalties|Restor3d: IP royalties|Smith & Nephew: Paid consultant|Total Joint Orthopedics: Paid consultant|Zimmer: research.

